# Second-Line Antiretroviral Treatment Outcome in HIV-Infected Patients Coinfected with Tuberculosis in Pakistan

**DOI:** 10.1155/2023/4187488

**Published:** 2023-04-19

**Authors:** Muhammad Shafiq, Sana Zafar, Aftab Ahmad, Abeer Kazmi, Alina Fatima, Tanvir Ahmed Mujahid, Rizwan Qazi, Nasim Akhter, Amir Shahzad, Saif Ur Rehman, Muhammad Adnan Shereen, Muhammad Zeeshan Hyder

**Affiliations:** ^1^Department of Biosciences, COMSATS University Islamabad (CUI), Park Road, Chak Shahzad, Islamabad, Pakistan; ^2^Services Institute of Medical Sciences, Lahore, Punjab, Pakistan; ^3^Department of Microbiology, Kohsar University Murree, Punjab, Pakistan; ^4^Institute of Hydrobiology, Chinese Academy of Sciences, University of Chinese Academy of Sciences (UCAS), Wuhan, China; ^5^Dermatology Department, Combined Military Hospital (CMH) Kharian, Punjab, Pakistan; ^6^Pakistan Institute of Medical Science (PIMS), Islamabad, Pakistan; ^7^Nishtar Medical University, Multan, Pakistan; ^8^Rahman Medical Laboratories, Kabul, Afghanistan

## Abstract

**Background:**

Tuberculosis (TB) coinfection in human immunodeficiency virus- (HIV-) infected patients is considered a risk of antiretroviral therapy (ART) failure. Coadministration of antitubercular therapy (ATT) with ART is another challenge for TB management.

**Objective:**

The study was aimed at investigating contributing factors affecting treatment outcomes in HIV-/TB-coinfected patients.

**Design:**

Cross-sectional. *Setting*. Samples were collected from the Pakistan Institute of Medical Sciences Hospital Islamabad. *Subject and Methods*. Clinicodemographic and immunovirological factors between the two groups were compared. The Student *t*-test and chi-square test were applied to compare outcome variables, and logistic regression was applied to determine the effect of TB on virological failure (VF). *Main Outcome Measures*. TB coinfection did not increase VF even in univariate (*p* = 0.974) and multivariate analysis at 6 and 12 months of 2^nd^-line ART start. ARV switching was significant (*p* = 0.033) in TB-coinfected patients. VF was significantly high in ATT-coadministered patients along with a viral load of ≥1000 (*p* = 0.000). *Sample Size and Characteristics*. We recruited seventy-four HIV patients on 2^nd^-line ART; 33 coinfected with TB were followed for at least 12 months.

**Conclusion:**

In HIV-/TB-coinfected patients, CD4 count, CD4 gain, and VF remained comparable to HIV patients with no TB infection. ATT significantly affects the treatment outcome, suggesting drug-to-drug interactions. These factors are important to revisit the therapeutic guidelines to maximize the benefit of dual therapy in resource-limited settings.

## 1. Introduction

Human immunodeficiency virus (HIV) infection in patients and tuberculosis (TB) coinfection in resource-constrained settings are a major concern. A lethal combination of two diseases is an emerging threat for healthcare providers. The risk of opportunistic infections (OIs), like TB, increases in HIV-infected patients because of suppressed immunity [1]. People living with HIV are 15-22 times more likely to develop TB than a person without HIV [2]. In 2020, an estimated 10 million new TB cases were reported worldwide. It is estimated that of these TB cases, 11.0% may be coinfected with HIV and about 215,000 people died of HIV-associated TB. Without proper treatment, nearly all HIV-positive people with TB will die [3]. In the general population, HIV prevalence is less than 1% in Pakistan but Pakistan is ranked fifth in the world for TB burden with 0.51 M new cases each year [3]. Endemic HIV has high prevalence in a subpopulation of male sex workers, female sex workers, and transgender and in intravenous drug users. It is estimated that in 2017, about 7,200 (3,600-12,000) HIV patients were coinfected with TB in Pakistan [4]. Lifelong antiretroviral therapy (ART) is the only therapeutic hope against HIV. Life expectancy is directly related to early detection of HIV and start of ART. All ART centers are providing free HIV treatment and care of patients in Pakistan.

Active TB (pulmonary or extra pulmonary) is also more challenging to detect in HIV patients. Tb progresses promptly in HIV patients. Coinfection of TB needs additional measures like antitubercular therapy (ATT). Coadministration of ART and ATT may decrease the effectiveness of each other and may result in premature discontinuation of therapy. On the one hand, HIV is acquiring drug resistance and a growing number of HIV patients on first line of ART require to shift on second line of ART. On the other hand, increasing prevalence of multidrug-resistant (MDR) and extremely drug-resistant (XDR) TB is an additional challenge for physicians to treat TB in general population and in HIV patients. Furthermore, therapeutic complications may arise due to human genetic factors involving ADME genes, adverse reactions (AR), drug-to-drug interactions, and a high number of pills leading to nonadherence to therapy [5]. These factors can lead to clinical and/or virological and/or immunological therapeutic failure in HIV-/TB-coinfected patients [6]. Limited data is available on therapeutic outcomes of ART in HIV-/TB-coinfected patients in underdeveloped countries and is scarce in Pakistan [7]. This study was conducted to understand factors responsible for therapeutic failure in HIV-/TB-coinfected patients and to formulate future policies and strategies regarding early detection of disease and evidence-based and individualized-based treatment [8].

## 2. Material and Methods

The study was conducted at HIV/AIDS Care and Treatment Center, PIMS, Islamabad, Pakistan. It is the only focal and referral center in the capital of Pakistan, Islamabad. HIV patients were divided into two groups. The first group of patients was designated as HIV^pos^TB^pos^, taking 2^nd^-line ART on initiation of treatment, and was TB coinfected (*n* = 33). The second group was designated as HIV^pos^TB^neg^, taking 2^nd^-line ART on initiation of treatment, and was without TB coinfection (*n* = 41). Two patients, who were treated for TB before the start of 2^nd^-line ART, were excluded. Characteristics of the two groups are compared in Table 1. HIV^pos^TB^pos^ was further subdivided into two groups. The first group ART^yes^ATT^yes^ is comprised of patients coadministered with ART and ATT. The second group ART^yes^ATT^no^ is comprised of HIV-/TB-coinfected patients only on ART.

Patients were requested to provide written consent for participation in the study. HEC Pakistan provided funding for the study. The ethical committee of CUI Islamabad approved the study (CIIT-BIO-Science/Office/215-2016) dated 10^th^ May 2019. All Pakistani adult patients that shifted to 2^nd^-line ART for at least one year after 1^st^-line ART resistance, between December 2005 and June 2020, were enrolled. All patients with complete clinical data, CD4, and viral load (VL) data on follow-up were included. Females who were pregnant and transgender were excluded. Real-time PCR-based, HI-Virus-1 RG-RT-PCR Kit was used to determine the VLon Rotor-Gene Q-PCR system (Qiagen, Germany) according to the manufacturer's instructions. BD FACSCalibur flow cytometry (Becton-Dickinson, USA) was used to determine CD4 count according to the manufacturer's instructions. Treatment failure is defined as advancement of disease even after initiation of 1^st^- or 2^nd^-line ART in terms of clinical, virological, or immunological failures which are defined as under. A definite diagnosis of treatment failure based on clinical and immunological failure criteria should be supported by virological failure. Clinical failure is defined as new or recurrent WHO stage IV event and also certain stage III conditions. A definite virological failure is when a single VL is >10,000 copies/ml at 12 months of follow-up. A probable virological failure is when either a single VL is >1000 copies/ml at 12 months or a VL at 12 months is ≥400 copies/ml, which is still elevated on second measurement taken after 30 days. Immunological failure is the decline of CD4 counts, less than the CD4 count before the start of treatment or <50% decrease of a peak value on 2nd-line ART or persistently lower than 100 cells/ml [9].

SPSS version 26.0 was used for statistical analysis. Categorical variables were described as frequency (%) and continuous variables as mean ± standard error of the mean (std. error mean). Two-tailed tests were performed and *p* < 0.05 was considered significant. Categorical outcome variables were compared using the chi-square (*χ*^2^) test. An independent-sample Student *t*-test was used for continuous variables. The effect of TB coinfection on virological failure was determined through binary logistic regression.

## 3. Results

Seventy-four patients contributing 209.33 person-years (p-y) of follow-up on 2^nd^-line ART were included in the analysis. A significant difference (*p* = 0.021) in mean age at the start of 2^nd^-line ART was recorded between HIV^pos^TB^pos^ and HIV^pos^TB^neg^ patients (Table 1). There was no other significant difference between the two groups at the start of 2^nd^-line ART start. Non-Tenofavir-based regimens (*p* = 0.941) were prescribed in 44.9% and 55.1% of patients in HIV^pos^TB^pos^ and HIV^pos^TB^neg^ patients, respectively (Table 2). HIV^pos^TB^pos^ patients experience at least one antiretroviral drug substitution (*p* = 0.033). Relationship status, employment status, education level, weight, and 1^st^-line nonnucleoside reverse transcriptase inhibitor regimen at 2^nd^-line ART start in both groups were insignificant (Table 1).

**Figure 1:**
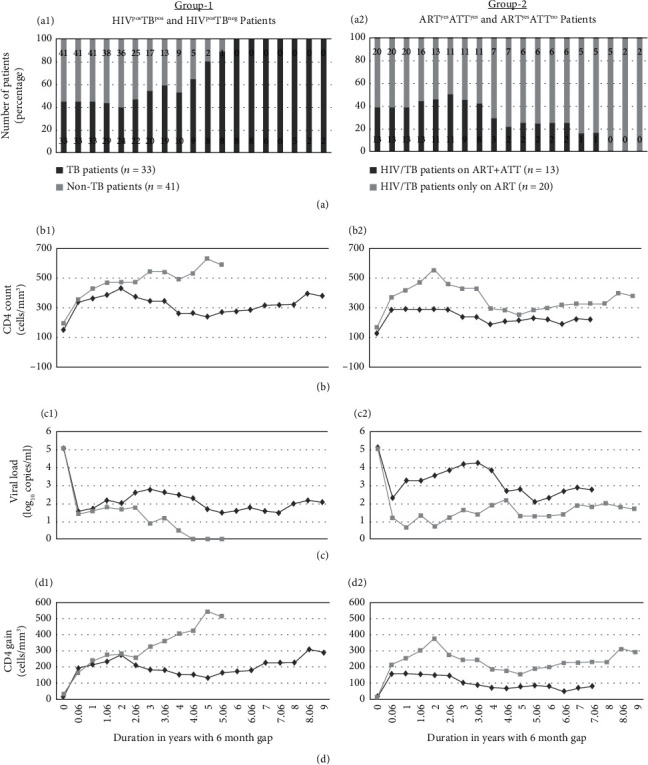
Virological and immunological outcome in TB coinfection and ATT coadministration in HIV patients on 2^nd^ line of treatment. HIV^pos^TB^pos^: HIV- and TB-coinfected patient group; ART^yes^ATT^yes^: antiretroviral therapy and antitubercle therapy-coadministered patient group; ART^yes^ATT^no^: only antiretroviral therapy-administered patient group. A1: describes percentage of HIV^pos^TB^pos^ and HIV^pos^TB^neg^ patients on follow-up for a period of nine years with 6-month gap in between. Numbers shown in bars are number of patients. A2: describes percentage of patients on ART^yes^ATT^yes^ and ART^yes^ATT^no^ follow-up for a period of nine years with 6-month gap in between. Numbers shown in bars are number of patients. B1: CD count (cell/mm^3^) of HIV^pos^TB^pos^ and HIV^pos^TB^neg^ patients during period of study. B2: CD count (cell/mm^3^) of patients on ART^yes^ATT^yes^ and ART^yes^ATT^no^ during period of study. C1: viral load (log_10_ copies per ml) of HIV^pos^TB^pos^ and HIV^pos^TB^neg^ patients during period of study. C2: viral load (log_10_ copies per ml) of patients on ART^yes^ATT^yes^ and ART^yes^ATT^no^ during period of study. D1: CD4 gain (cell/mm^3^) of HIV^pos^TB^pos^ and HIV^pos^TB^neg^ patients during period of study. D2: CD4 gain (cell/mm^3^) of patients on ART^yes^ATT^yes^ and ART^yes^ATT^no^ during period of study.

No significant difference in CD4 count, CD4 gain, and VL was observed after 6 months and 12 months of 2^nd^ line of ART in both groups (Figure 1). Similarly, clinical outcome and virological failure (VF) at census were also not significantly different in both groups. Effect of TB coinfection on VF, using logistic regression analysis, showed no significant difference at 6 and 12 months of infection both in univariate (*p* = 0.924) and multivariate (*p* = 0.081) analyses (Table 2).

Concomitant use of ATT is significantly associated with VF (*p* = 0.005) and VL of ≥1000 copies/ml after 12 months of 2^nd^-line ART treatment (*p* = 0.000). Adherence of <95% (*p* = 0.000) was significantly associated with VF in ART^yes^ATT^yes^ patients. Mortality rate was 3.17 (95% CI 0 to 5.94) per 100 p-y with 12.16% (*n* = 9) deaths in both groups. Mortality was not associated with adherence as 60% of dead patients were having ≥95% of adherence. No significant difference in mortality rate was observed between the two groups and even in patients on ATT along with ART (Table 3).

## 4. Discussion

TB coinfection and simultaneous use of ATT are known global factors resulting in ART failure [10], and it becomes more important in Pakistan, because our country is at the 5^th^ position among high-TB-prevalence countries [11, 12]. The prevalence of HIV-TB coinfection is slowly evolving in the Asia-Pacific region. It is estimated that 17.2% of HIV patients are coinfected with TB [13]. In our study, among 44.6% HIV-coinfected patients with TB, 39.4% (*n* = 13) were coadministered with ATT along with 2^nd^-line ART.

During treatment, patients experience different OIs, which are tabulated in Table 1 . Difference of OIs even between the two groups is insignificant (*p* = 0.064). However, candidiasis and chronic diarrhea were experienced by most patients. The exact cause of diarrhea could not be determined due to a lack of experienced microbiological testing in routine laboratories as reported by other local studies in which cryptosporidium-related diarrhea was also underreported [7, 14].

The mortality rate was 13.51% (*n* = 9) in both groups. Rates of mortality varied substantially, ranging from 11% to 29% in different studies [8]; however, in most settings, coinfection of TB in HIV patients is the most common cause of death [15]. Difference in mortality rate can be multifactorial: suboptimal therapeutic and clinical management of patients, ineffectiveness of ATT regimen provided, prevalence of drug-resistant TB in the region, unavailability of TB drug resistance testing, duration of ATT treatment provided to patients, and patient adherence to therapy [8]. There was no significant association of VF with age groups, but 88.9% (*n* = 8) deaths were observed in association with age > 30. However, with better care, diagnosis, and therapeutic management, the mortality rate has declined significantly among HIV-/TB-coinfected patients in the Asia-Pacific region since 2004 [13]. Even then, 0.4 M deaths among HIV-/TB-coinfected patients were reported in 2016 [2] and it is also observed that the motility rate was high in HIV-/TB-coinfected patients on 2^nd^-line ART [16].

CD4 count, CD4 gain, and VL were not significantly different in patients with HIV-/TB-coinfected patients. CD4 count and CD4 gain were not significantly different either in patients on ART, coadministered with ATT. But VL was significantly suppressed in HIV-/TB-coinfected patients, not on ATT, as compared to patients on ATT. A possible interaction between ATT and 2^nd^-line ART could be suggested. Nevertheless, it is believed that patients on ATT could not adhere to ART and hence, immunological response will be worsened [1]. Coadministration of ART and ATT was linked to suboptimal adherence to treatment, and it could be related to the outcome of ART [17, 18]. According to an African study, 2^nd^-line VF was strongly associated with concomitant TB treatment along with a lower level of adherence. Mechanisms behind the VF after 2^nd^-line ART in HIV-/TB-coinfected patients need to be explored [19].

TB was not significantly associated either in the univariate analysis (*p* = 0.924) or in the multivariate model (*p* = 0.081) with VF. TB was included in the multivariate model due to its close association with HIV [20]. However, the coadministration of ATT with 2^nd^-line ART is significantly associated with VF. This is the first study, analyzing the treatment outcome of coadministration of ATT and 2^nd^-line ART in Pakistan. Baqi et al. described the interaction of 1^st^-line ART and ATT, especially with rifampicin. Efavirenz has good therapeutic response than nevirapine in coadministration with ATT [5, 7]. In our institution, 1^st^-line ART regimen with efavirenz was the preferred therapy and was predominantly prescribed in our study group. Protease inhibitors (PIs) with other ART combinations and ATT may have a role to interact with each other. Another factor could be the genetic variability effect of ADME (absorbance, distribution, metabolism, and excretion) genes [19]. This interaction could be compensated by a high dose of PIs, which may result in adverse effects on the patient or the patient may exhibit nonadherence due to an increase in dose and number of pills, posing additional risks to therapeutic failure [21]. Treatment outcomes in HIV-/TB-coinfected and ART/ATT-coadministrated patients suggest drug interactions that warrant further pharmacokinetic and pharmacogenomics studies in lieu of genetic makeup of the Pakistani population. Concomitant use of ATT with 2^nd^-line ART needs more optimization in the country. Ineffective ATT in HIV-/TB-coinfected patients is leading to multidrug-resistant (MDR) TB which further complicates the therapeutic management [22]. Integration of TB and HIV control services on a national level needs time [23]. National health authorities have started taking action accordingly by merging malaria, TB, and HIV national programs but this needs to be done on a provincial level too.

In our institution, we have been using WHO-recommended ritonavir boosting dose with lopinavir, concomitantly in combination with rifampicin. But this combination has resulted in hepatotoxicity and poor tolerability [21]. On the other hand, raltegravir with efavirenz, or the use of rifabutin, is preferred when ritonavir-boosted PIs are used as 2^nd^-line ART [24]. National programs are suggested to revise therapeutic guidelines and to ensure the availability of rifabutin and raltegravir in Pakistan.

## 5. Conclusion

In HIV-/TB-coinfected patients, CD4 count, CD4 gain, and VF remained comparable to HIV patients with no TB infection. ATT significantly affects the treatment outcome, suggesting drug-to-drug interactions. These factors are important to revisit the therapeutic guidelines to maximize the benefit of dual therapy in resource-limited settings. A small sample size is a limitation but important where low numbers of patients are on 2^nd^ line of ART. Drug-to-drug interactions and plasma levels were not determined. TB coinfection did not significantly increase the risk of virological failure. On the other hand, coadministration of ATT with ART significantly increases the virological failure. It is mandatory for our national AIDS and TB control program to scale up concurrent HIV/TB treatment under integrated care.

## Figures and Tables

**Table 1:** Characteristics of HIV patients on 2^nd^-line ART.

S. No.	Characteristic/risk factor	Total patient No. (%) *N* = 74	HIV^Pos^TB^Pos^ patient No. (%) 33 (44.6%)	HIV^Pos^TB^Neg^ patient No. (%) 41 (55.4%)	*p* value
1	Gender				
	Female	19 (25.7%)	6 (31.6%)	13 (68.4%)	—
	Male	55 (74.3%)	27 (49.1%)	28 (50.9%)	0.186
2	Relationship status				
	Married	50 (67.6%)	26 (52.0%)	24 (48.0%)	—
	Single	24 (32.4%)	7 (29.2%)	17 (70.8%)	0.064
3	Employed				
	No	37 (50%)	17 (45.9%)	20 (54.1%)	—
	Yes	37 (50%)	16 (43.2%)	21 (56.8%)	0.815
4	Education level				
	Illiterate	29 (39.2%)	12 (41.4%)	17 (58.6%)	—
	<10 years of education	36 (48.6%)	17 (47.2%)	19 (52.8%)	0.906
	≥10 years of education	9 (12.2%)	4 (44.4%)	5 (55.6%)	0.895
5	Weight at 2^nd^-line ART start (kg) mean ± std.error of the mean				
		—	54.55 ± 2.3	52.54 ± 2.4	0.557
6	1^st^-line regimen NNRTI^∗^ initiated				
	Efavirenz based	51 (68.9%)	23 (45.1%)	28 (54.9%)	—
	Nevirapine based	23 (31.1%)	10 (43.5%)	13 (56.5%)	0.897
7	Age at start of 2^nd^-line ART (year) mean ± std.error of the mean				
		—	38.6 ± 1.9	31.7 ± 2.1	0.021
8	Clinical WHO stage at 2^nd^-line ART start				
	Stages 1 & 2	55 (74.3%)	22 (40.0%)	33 (60.0%)	—
	Stages 3 & 4	19 (25.7%)	11 (57.9%)	8 (42.1%)	0.176
9	Coinfection with hepatitis B & C				
		10 (13.5%)	7 (70.0%)	3 (30.0%)	0.082
10	CD4 count at 2^nd^-line ART start (cells/mm^3^) mean ± std.error of the mean				
		—	153 ± 20	193 ± 25	0.216
11	VL at 2^nd^-line ART start (log_10_ copies/ml) mean ± std.error of the mean				
		—	5.1 ± 0.2	5.1 ± 0.1	0.928

^∗^NNRTI: nonnucleoside reverse transcriptase inhibitors.

**Table 2:** Clinico-immunological and virological outcome in HIV patients on 2^nd^-line ART coinfection with or without TB.

S. No.	Characteristic/risk factor	Total patient No. (%)	HIV^Pos^TB^Pos^ patient No. (%)	HIV^Pos^TB^Neg^ patient No. (%)	*p* value
1	Adherence to 2^nd^-line ART				
	<95%	15 (20.3%)	6 (40.0%)	9 (60.0%)	—
	≥95%	59 (79.7%)	27 (45.8%)	32 (54.2%)	0.688
2	NRTI^∗^ combination in 2^nd^-line regimen initiated				
	Tenofavir based	49 (66.2%)	22 (44.9%)	27 (55.1%)	—
	Non-Tenofavir based	25 (33.8%)	11 (44.0%)	14 (56.0%)	0.941
3	ARV^∗^ side effect during 2^nd^-line ART				
		13 (17.6%)	8 (61.5%)	5 (38.5%)	0.176
4	ARV switching during 2^nd^-line ART				
		9 (12.2%)	7 (77.8%)	2 (22.2%)	0.033
5	Opportunistic infections other than TB				
		50 (67.6%)	26 (52.0%)	24 (48.0%)	0.064
6	Clinical outcome				
	Death	9 (12.2%)	6 (66.7%)	3 (33.3%)	—
	LTFU	1 (1.4%)	1 (100%)	0	—
	Alive	64 (86.5)	26 (40.6%)	38 (59.4%)	0.180
7	Virological failure while on 2^nd^-line ART				
		29 (39.2%)	13 (44.8%)	16 (55.2%)	0.974
8	CD4 at 12 months of 2^nd^-line ART (cells/mm^3^) mean ± std.error of the mean				
	Total	—	368 ± 44	428 ± 43	0.334
	<200	14 (18.9%)	6 (42.9%)	8 (57.1%)	—
	≥200	60 (81.1%)	27 (45%)	33 (55%)	0.885
9	CD4 gain after 2^nd^-line ART of 12 months (cells/mm^3^) mean ± std.error of the mean				
	Total	—	217 ± 38	233 ± 33	0.741
	<100	19 (25.7%)	7 (36.8%)	12 (63.2%)	—
	≥100	55 (74.3%)	26 (47.3%)	29 (52.7%)	0.303
10	VL after 2^nd^-line ART of 12 months (copies/ml) mean ± std.error of the mean				
	Total	—	204,979 ± 97,941	340,077 ± 186,225	0.551
	<1000	52 (70.3%)	23 (44.2%)	29 (55.8%)	—
	≥1000	22 (29.7%)	10 (45.5%)	12 (54.5%)	0.923

^∗^NRTI: nucleoside reverse transcriptase inhibitors; ARV: antiretroviral; LTFU: loss to follow-up.

**Table 3:** Clinico-immunological and virological outcome in HIV-/TB-coinfected patients on 2^nd^-line ART, coadministered with or without ATT.

S. No.	Characteristic/risk factor	Total patient No. (%)	HIV^yes^TB^yes^ patient on ATT No. (%)	HIV^yes^TB^no^ patient not on ATT No. (%)	*p* value
1	Adherence to 2^nd^-line ART				
	<95%	6 (18.2%)	3 (50.0%)	3 (50.0%)	—
	≥95%	27 (81.8%)	10 (37.0%)	17 (63.0%)	0.000
2	NRTI combination 2^nd^-line regimen initiated				
	Tenofavir based	22 (66.7%)	9 (40.9%)	13 (59.1%)	—
	Non-Tenofavir based	11 (33.3%)	4 (36.4%)	7 (63.6%)	0.801
3	ARV side effect during 2^nd^-line ART				
		8	7	1	0.000
4	ARV switching during 2^nd^-line ART				
		7 (21.2%)	3 (42.9%)	4 (57.1%)	0.833
5	Clinical outcome				
	Death	6 (18.2%)	3 (50.0%)	3 (50.0%)	—
	LTFU	1 (3.0%)	1 (100%)	0	—
	Alive	26 (78.8%)	9 (34.6%)	17 (65.4%)	0.355
6	Virological failure while on 2^nd^-line ART				
		13 (39.4%)	9 (69.2%)	4 (30.8%)	0.005
7	CD4 at 12 months of 2^nd^ line (cells/mm^3^)				
	<200	6 (18.2%)	3 (50.0%)	3 (50.0%)	—
	≥200	27 (81.8%)	10 (37.0%)	17 (63.0%)	0.557
8	CD4 gain after 2^nd^-line ART of 12 months (cells/mm^3^)				
	<100	7 (21.2%)	4 (57.1%)	3 (42.9%)	—
	≥100	26 (78.8%)	9 (34.6%)	17 (65.4%)	0.279
9	VL after 2^nd^-line ART of 12 months (copies/ml)				
	<1000	23 (69.7%)	4 (17.4%)	19 (82.6%)	
	≥1000	10 (30.3%)	9 (90.0%)	1 (10.0%)	0.000

^∗^NRTI: nucleoside reverse transcriptase inhibitors; ARV: antiretroviral; LTFU: loss to follow-up.

## Data Availability

All the data is available in the manuscript.

## Abbreviations

HIV: Human immunodeficiency virus
TB: Tuberculosis
ART: Antiretroviral therapy
ATT: Antitubercular therapy
VF: Virological failure
OIs: Opportunistic infections
WHO: World Health Organization
MDR: Multidrug resistant
XDR: Extremely drug resistant
AR: Adverse reactions
PIs: Protease inhibitors.
